# Art in Corporate Governance: a Deweyan Perspective on Board Experience

**DOI:** 10.1007/s40926-020-00152-y

**Published:** 2020-11-09

**Authors:** Donald Nordberg

**Affiliations:** Executive Business Centre, Bournemouth University, 89 Holdenhurst Road, Bournemouth, BH8 8EB UK

**Keywords:** Boards of directors, Corporate governance, Aesthetics, Art, Dewey

## Abstract

Corporate governance sits at the intersection of many disciplines, among them law, business, management, finance, and accounting. The point of departure for large portions of this literature concerns the ugliness of greed, ambition, misdemeanors, and malfeasance of corporations, their directors, and those actors who hold shares in them. This essay takes a rather different starting point. Drawing upon insights from a distant field, it uses the discussion of aesthetics in Dewey’s treatise on art to ask what motivates directors to act in ways that constitute the attention and engagement that we associate with the effectiveness of boards. Using Dewey’s thinking about aesthetic experience, this paper examines the experience of organization boards, both in the literature and in the personal experience of the author. These observations point to need to reflect on motivation when considering both the practice of corporate governance and the policy frameworks in which it operates.

## Introduction

The oak-paneled walls of the stately boardroom of one company where I worked were covered with large canvases painted by a variety of modern artists: impressionists, post-impressionists, and expressionists. The plain, plasterboard walls of boardroom of a charity I serve are lined with more modest yet no less evocative works: paintings, mainly on paper, by some of the people we help with their learning difficulties, mental health issues, and dementia.

The first, to be sure, was an ostentatious display for the few allowed to enter this special place. The second shows a need to brighten up the grim physical space, which doubles on other days as a meeting room in frequent use by staff from several departments. Yet these gestures also suggest that organizations – or more specifically the evolving groups of people who lead them – choose to remind themselves, and those who will follow them, of the larger things in life, outside the immediate issues needing decisions, and that the directors themselves like to be reminded of them.

Although the subject of, or analogies to, aesthetics rarely come up in writing on corporate governance, occasionally we find references to art in scholarly writing about management and leadership (Strati 1999; Ladkin 2008; Adler and Delbecq 2018). This essay uses the work of John Dewey to stretch such discussion into the boardroom. It wonders, how might board deliberations present something other than the problem of the self-centered, self-important agent we hear so much of in the literature of corporate governance? How might the forms of corporate governance conducted in these rooms be, well, beautiful?

Corporate governance can be ugly. At the top of corporations, greed seems prevalent and stewardship lacking; hostilities erupt around contested takeovers, vulture capitalism, and activist hedge funds instigating disruption. Other types of organizations, too, are replete with interpersonal rivalry, pay excesses and self-serving managers, and a sense of a need to win at the expense of other organizations that would eat our lunch. Such phenomena have generated considerable academic study – drawn from accountancy, financial economics, sociology, and (the lack of) business ethics. Much of it seeks to diagnose the causes from the symptoms of such corporate malaise and inform regulation and codes of conduct in the hope of curing the problem.

After sketching the history of corporate governance and the problems and purposes of boards, let us consider the argument of the pragmatist philosopher John Dewey that art can best be understood when viewed not as an object, but instead as an experience – of both the artist and the audience – and that experience invests meaning in activity. His theorizing took place in the intellectual context of the disruptions in trade, society, and politics of the 1930s and the Great Depression, ugly events that also spawned public concern for corporate governance.

Then we look at how such theorizing lets us reflect on the work and motivation of directors, drawing evidence from a combination of experience depicted in scholarly studies of boards, reports based on board observation, and my own experience of acting as a director. This essay highlights in the work of directors the possibility of a heightened sensibility akin to that of aesthetic experience, which may account for a part of director motivation that the corporate governance literature has largely overlooked. It concludes with observations about how those insights about what does right in boardrooms can help us better understand where they go wrong, and what directors, policymakers and the public at large can expect to achieve.

## Governing: The Purpose and Problems of Boards

The study of corporate governance largely arose from concern about problems with corporations and the people who direct them. Boards are seen as mechanisms of governance, to control the agency problem arising when managers work for distant owners (Fama and Jensen 1983; Jensen and Meckling 1976), or to mediate transaction costs hierarchically, rather than through markets (Williamson 1996; Blair and Stout 1999). Such considerations assume directors are governance instruments; they dominate empirical study, despite often ambiguous empirical findings (Bosse and Phillips 2016) and theoretical misgivings (Roberts et al. 2005).

The problems of corporate governance trace their roots to the Wall Street Crash of 1929 and the Great Depression that followed. Berle and Means (1932/1991) argued that widespread corporate failure in the years leading up to the crash arose from the increasing separation of corporate owners from the control of the corporation. It provided the base of agency theory.

Coase (1937) then suggested that the purpose of companies was to internalize transactions that markets could not efficiently conduct, creating the basis of transaction cost economics. A different view of corporate purpose arose in that time as well: that private enterprises, rather than the state, bring value to society through innovation and entrepreneurship. It is rooted in the thinking of Schumpeter, who located social good and not just social irresponsibility in corporate enterprise (published later as Schumpeter 1942/1976). Here, the creators are the entrepreneurs, who are not necessarily the owners.

These seminal works outlined the problems in capitalism and sought solutions that could defend it against a socialist or communist alternative. Grounded in these ways, the so-called theory of the firm explained how – despite the Crash – corporate entities bring about economic growth and ultimately societal value. And far from being dismantled as a hazard to society, corporations flourished, became more central, more important in western economies, and more dominant in the lives of people. Success also bred excess, in executive pay, diversions of corporate resources for personal gain, and recurrent cases of corporate collapse (MacAvoy and Millstein 2003; Nordberg 2020).

Scholars have sought to identify ways to prevent corporate malfeasance, identifying board-focused inputs, like board composition or director independence, that might deter corporate excess and improve corporate outcomes (e.g. Payne et al. 2009). Such analyses yielded ambiguous results (Dalton and Aguinis 2013; Daily et al. 2003), yet practitioners – directors and the consultants who serve them – often find in them something helpful. Policymakers have sought to use them to design structures and mechanisms in the pursuit of best practice, which inform legal and regulatory moves to constrain corporate managements and boards, or codes of conduct to guide boards toward safer outcomes. This is often called the “control” role of boards.

The corporate governance literature identifies a second role for boards, however, one of “service” (Hillman and Dalziel 2003). This view sees directors facilitating access to resources, with outside, non-executive directors helping managers through boundary-spanning activities and contributions to strategic decisions.

While valuable in identifying and potentially solving the problems of governing organizations, such economics-focused analysis overlooks an element that the practitioners I know see as important: the pleasure in, and the sensation of, creating value. It is a sense that directors and senior managers often seem to derive from the processes of board decision-making. The work directors seem most proud of is fending off disasters, thwarting attacks, and preventing the departures of key personnel. Quiet accounts of directors are brightened by expressions of a job well done, even if the job done leaves no trace visible to the outside world, which may not even be recorded in the confidential minutes of board meetings. Such achievements rarely hit the headlines or form the basis of an entry or black mark on a director’s *curriculum vitae*. They defy observation, let alone measurement of the efficiency in process or the success in terms of outcomes.

The director’s role we call “outside” in America or “non-executive” in Britain comes with symbolic and pecuniary rewards, that is, the status of being “director” or an income for seemingly little work but also at some risk. At large companies, directors give their advice for levels of pay comparable to top management consultants. In smaller firms, the rewards are less generous; in charities, directors make similar time commitments, but most go without pay. And, unlike shareholders, who can lose only as much (or little) as they invest, the directors those investors elect to serve them face unlimited, personal liability when decisions go wrong, protected in part by insurance. Consultants can often retreat unnoticed from failed projects. Directors risk being prohibited from serving again on boards.

Against such a background, why would someone want to be a company director? This paper asks a narrower version of that question: If motivation includes the sense of satisfaction that directors derive from their work, how does it arise? It seeks an answer by drawing upon theory developed at great distance from the disciplines that usually inform writings on boards of directors.

## Art, Experience, and the Great Depression

In his 1930s lectures at Harvard University in memory of William James, the American philosopher and polymath John Dewey laid out a theory of aesthetics, subsequently published as *Art as Experience* (1934/1958). The date of delivery and publication is pertinent. This was a few years after the Wall Street Crash, the first global corporate governance crisis, when thinkers from a wide range of fields were trying to understand what had gone wrong.

On the surface, the subject of Dewey’s essay could scarcely be more remote from the corporation and its economics. He writes on painting, sculpture, architecture, the novel, and poetry, and how both the artist and the audience draw meaning from the experience of the artistic creation. His depiction of the aesthetic has little to do with narrow conceptions of “high art.” Dewey was an early advocate of the aesthetics of jazz, which many high-brow critics of the time dismissed as noise. He writes that to understand the aesthetic, “one must begin with it in the raw … the sights that hold the crowd – the fire engine rushing by; the machines excavating enormous holes in the earth” (Dewey 1934/1958). These are examples of the sensation of the observer. A little later he writes, “The intelligent mechanic engaged in his job, interested in doing well and finding satisfaction in his handiwork, caring for his materials and tools with genuine affection, is artistically engaged”; this depicts the creator’s response. In contemporary as well as ancient societies, practices including body scarification, ornamental dress and jewelry indicate that “everything that intensifies the sense of immediate living in an object of intense admiration” (1934/1958).

He rejects theories of art that spiritualize it “out of connection with the objects of concrete experience.” Art is “the living and concrete proof that man is capable of restoring consciously, and thus on the plane of meaning, the union of sense, need, impulse and action.” With these observations, Dewey brings us a range of objectives that art serves but not quite a definition of art as experience. We have “*an* experience when the material experienced runs its course to fulfillment” (1934/1958, emphasis in the orginal).

This assertion is a definition even his supporters say is incomplete and perhaps paradoxical. In a book largely honoring Dewey, Shusterman (2000) calls his “clearly an inadequate and inaccurate definition”. In Dewey’s defense, Shusterman asserts that this approach was, at least, not part of the “long history of failed attempts at essentialist definitions of art” (2000). Dewey did not attempt to articulate the essence of art, which would have defied the nonfoundationalist core of his pragmatist philosophy. Instead, he calls attention to the pragmatists’ view of the centrality of practice. For Dewey, artistic sensation arises not in the object, the thing created, but rather in the experience of creating and then observing it. The heightened sensitivity, the intensification of the immediate, achieved in the experience of making or apprehending the aesthetic object is what signifies the artistic.

On this view, art-as-experience implies something historically situated, enduring but impermanent, reflective of and dependent on context, with meaning created from the interaction of the creator and then audience with the thing created. This is not an aesthetics of ideal types, of striving for perfection. The experience of the artist will differ from that of the audience. Yet both will deem their experience as art when they encounter enhanced sensitivity and sensibility, that is, when the experience brings new sensations, cognitive and emotional. Moreover, apprehension – by creator and observer – of routine, imitative, mass-produced objects may not achieve such feelings, producing instead something that might be termed, as Smuts (2005) argues, an “anesthetic experience.”

For Dewey, aesthetic experience has a moral dimension, a proposition he argues through the negative. “One great defect in what passes for morality is its anesthetic quality. Instead of exemplifying wholehearted action, it takes the form of grudging piecemeal concessions to the demands of duty” (1934/1958). When the artist or the audience focuses on duty to some external standard their sensitivity can be blunted. Turning the argument around, he argues that, for the Greeks, moral action involves conduct that is elegant, in proportion, and graceful. Practical activity, he writes, has an aesthetic quality when it is “integrated and moves by its own urge to fulfillment.”

Dewey links this fulfillment to the artist’s “unusual sensitivity to the qualities of things” (1934/1958), as artists shape and reshape their objects until they are satisfied with what they perceive in them. The audiences of art – the beholders, in Dewey’s argument – perceive the object by creating their own experiences of it, perceptions comparable to but necessarily not identical with those of the artist.

This experiential view of art, related to but separate from the object, can apply to performance arts: to drama, which is enacted and then is no more; to improvisational jazz, which lacks even a script; and to art installations, in which the thing experienced is in part the experiences of other members of the audience. Let us draw an analogy, then, and consider how we can think of non-things – management and leadership, for example – as beautiful or ugly, as well as mundane or dull. And if art lies not so much in the tangible object but in the experience, the term might justifiably be used to signify the experience of the intangible as well.

## The Art of and in Corporate Governance

Because the corporate governance literature focuses on organizational issues, accounts of director experiences are rare. Concerns that dominate include the search for solutions to the “agency problem” of self-serving managers, in which boards are mechanisms and directors instruments (Kumar and Zattoni 2018). The quest for stewardship by company directors and top managers takes us theoretically into the realm of psychology (Davis et al. 1997), though with the intent of identifying paths to better firm performance. Studies that penetrate the fabled “black box” of the boardroom (Zona and Zattoni 2007) tend to examine processes and practices like strategy work (Concannon and Nordberg 2018; Machold et al. 2011; McNulty and Pettigrew 1999), rather than director motivations.

There are occasional glimpses of attitudes and feelings, however. Reflecting on her investigations of board work, involving repeated interviews over a decade with individual directors, Pye (2002) reaches into the language of art to assert that “it is impossible to define an ideal culture, particularly where the tone and tenor of relationships amongst board members influences conduct.” Samra-Fredericks (2000) dissects the validity claims of directors and top managers in discussion and how telling a strategy “story” in a way that “others have to believe it” provides a “feel good factor.”

The autoethnographic work of Parker (2007a, 2007b) documents the inner workings of the boards of professional organizations. He describes how individual directors champion causes, formally and informally, to command attention of the others and how behavior differs depending on the assertiveness of the CEO. Mutual respect provides a “social lubricant” and permitting contentious issues to be discussed with “informality and humour” (Parker 2007b).

In another autoethnographic account, Gibbon (2012) reaches for metaphor – of a garden and a jigsaw puzzle – to record her sense of satisfaction concerning the “uncertain, complex and messy” nature of accountability in the setting of a board of a nonprofit organization. She reflects that the garden metaphor may mislead by suppressing certain features of the experience, the way “weeds, pests and unexpected weather conditions to reveal a different perspective.” As close as these come to showing us the inner workings of boards, they remain descriptive of behavior or reflective of reaction to processes. They do not quite capture how the directors found expression in the boardroom or how they felt about the expression of others.

How, then, might we employ Dewey’s concept of art-as-experience to understand the work of corporate directors? Dewey’s treatise does not offer a handy template to assess its application to other fields; indeed, imagining that it might could be antithetical to his argument. Instead, let us consider here, tentatively, the elements of the work of boards of directors, how they might constitute the “act of expression” (Chapter IV), the iterative construction and reconstruction involved in creating a “union of sense, need, impulse and action” and of practitioners “finding satisfaction” in their “handiwork” (1934/1958), expressions Dewey uses to describe the experience of creation. Then let us consider, even more tentatively, how observers of board meetings – the middle-ranking executives invited to present to the board, or new directors finding their way into discussions of longstanding, gnarled problems – finding meaning through the experience.

To do so, I draw in part upon some of the less fully articulated aspects of qualitative studies of boards in operation. To overcome the thinness of the data, I draw as well on personal reflections of the boards on which I have served, an unconventional approach but one with parallels in the reflection that underpins practitioner accounts that occasionally appear in academic journals on management, as well as much philosophical writing. Of the boards I served, two were small, private firms in publishing, which sought capital for expansion and then proved unable to use it to good effect. Two others were large charities, with income in the millions and tens of millions of pounds sterling, each with active and engaged boards.

Board meetings of one of the publishing companies were infrequent, acrimonious, and unproductive. The company limped on for several years before collapsing. The other had frequent and full meetings, leading to collective decisions the participants found satisfying. But the company’s financial circumstances were far more fragile than we thought, which led to its ultimate failure. The charities both dealt with difficult finances – not-for-profit organizations rarely achieve the financial buffer to guarantee viability – but board meetings were productive, insightful, and enlightening, for managers and directors alike. In early 2020, both charities faced potential existential threats in the coronavirus and covid-19 pandemic.

## The Act of Expression

For Dewey, expression involves more than an explosion of emotion. For example, he draws a contrast between self-expression and self-exposure; the latter involves disclosing character to others in a way that is “only a spewing forth” (1934/1958). Dewey (1934/1958) describes the act of expression as arising from an “impulsion.” He draws a distinction to an impulse, which is “specialized and particular.” Impulsion is a movement outward and forward of the whole organism, like a craving for food. Impulsion alone is not sufficient for expression, however. When impulsion meets resistance, the person is forced into reflection, a “transformation of energy into thoughtful action”; “emotional discharge is a necessary but not a sufficient condition for expression” (Dewey 1934/1958).

In a study for a professional body, Kakabadse et al. (2017) report on a company chairman, who remarked: “I used to talk about boards as being like an orchestra. You want different instruments who together make, not a discordant noise, but a harmonious noise. Not too harmonious.” Here, we can see in action the distinction Dewey makes between self-expression and self-exposure. Being “not too harmonious” is set against not just harmony, but also “discordant noise”; it signifies the boardroom equivalent of musical complexity. Discordant noise on its own is, in Dewey’s term, self-exposure: emotional discharge in disregard of others.

The lack of expression is captured by Langevoort (2001), who writes of boards where the outside, non-executive directors have been captured by the CEO. They become mere social clubs with a rubber stamp to approve the CEO’s decisions. Sonnenfeld et al. (2013) found that many chief executives expressed disappointment in outside directors who failed to provide the resistance that leads to reflection. Such experience might be described as artless, anesthetic corporate governance (Cf. Cohen 2007).

My experience of boards has examples of the anesthetic. At board meetings, when the executives were well prepared even if the non-executives were rather less so, decisions passed easily and efficiently; executives were content with the outcomes; yet no one felt proud of their work. By contrast, board meetings in which challenge took place, during which both executives and non-executives learned more about the business and its problems, led to a sense of satisfaction, even delight, at the experience. Strangely, that sensation arose even in circumstances where the decisions were not universally welcomed and did not resolve the problems those boards were trying to address. What mattered was that the encounters increased awareness of the complexities.

For example, in a social-care business, where the sources of income – local government agencies who are the “customers” – hold all the power, boards have few levers to improve performance. But we came to realize that through what Dewey might call each director’s “impulsion” to make things better, which met resistance: Some directors backed alternative approaches with considerable vigor but then backed away as greater insight into the power relationships became clear and a partial consensus emerged. Such debate creates the heightened sensitivity to complex of context and resources, of opportunities and limitations. This discord within harmony is akin to artistic expression.

Within expression, Dewey (1934/1958) notes four characteristics of art: a) its creation of an integral experience, in which b) the object comes about through pressure on natural impulses, implying c) that construction requires time, not instantaneous emission, and d) excitement in production stirs up the store of attitudes and meanings. Let us look at each in turn.

### Integral Experience

For Dewey, an integral experience arises from the “interaction of organic and environmental conditions and energies” (1934/1958). He leaves this depiction rather vague, but the argument points to the substance of the artist (“organic”) interacting with the surrounding materials and circumstances. It seems to indicate the importance of what one of Dewey’s most influential followers terms “contingency” (Rorty 1989). In the setting of boards, the experience of integration comes as we build a picture of the business as a whole and how it aligns with the contingencies of the business environment, reacting or failing to reaction to changing circumstances.

Some studies have sought to capture the ethos of the boardroom and its interaction through the comments that directors make to consultations about the codification of board practice. Spira and Slinn (2013) give an account of the formulation of the Cadbury Code in the UK, which cites in part comments from corporate directors about the negative impact the code might have on the candor of board discussion, pitting non-executives directors against the executives. They feared the experience of board work would disintegrate. Nordberg (2017) extends that by examining how directors sought to preserve a version of board ethos through the drafting of three major versions of the UK code over two decades, and their fear of a loss of intensity of interaction. In both accounts, we hear corporate directors arguing that the constraints proposed will stifle open discussion and drive a wedge between directors engaged in complex relationships. Advocates of constraints suggest that is the point of codes: to prevent cozy relationships from forming that might lead to less stringent monitoring of managerial behavior. These are, respectively, arguments for and against the integral/organic stance, against and for rules to replace contingency.

In my cases, both publishing businesses involved creative individuals with a store of knowledge and the ability to project it into words and images in print. Both faced the challenge of the sudden explosion of the internet, however, which was replacing print publications and opening new avenues that had little to do with the skills resident “organically” in the companies. Acrimonious board meetings took place at one of them, when management refused even to discuss how the business idea might be translated into a different medium. At the other, the while the problem was recognized, the skills deficit of the incumbents, and the incumbents’ voting power, we failed to resolve the issues. Such meetings were unsatisfying in their outcomes, but they nonetheless yielded greater appreciation of the issues among all the directors and a degree of satisfaction among directors. Whatever the quality of the experience, the lack of tangible progress at both firms had consequences. As opportunities were debated but left for others to pursue, finances grew tight and pressure mounted.

### Pressure and Time

For Dewey, the experience of pressure and time are deeply linked:

The thing expressed is wrung from the producer by the pressure exercised by objective things upon natural impulses and tendencies – so far is expression from being the direct and immaculate issue of the latter.… The act of construction in time that constitutes a work of art is a construction in time, not an instantaneous emission (Dewey 1934/1958).

Expression is thus mediated. Artists use tools to shape the raw materials and mental effort to shape the ideas and emotions. They follow impulses but also employ pre-existing templates. Art has its genres as well as tools that infer imitation and bring the legitimacy of recognition. The artist then constructs the object and reconstructs it through revision and iteration. These are the changes a painter makes while making sense of the thing observed and the ideas that come to mind, or the revisions a writer makes in crafting a manuscript and finding the right words. Dewey holds that “expression of the self in and through a medium … is *itself* a prolonged interaction.” Both the self and the medium “acquire a form and order they did not at first possess” (1934/1958, emphasis in the original).

In board work, iteration to achieve meaning takes a different form. There are internal pressures, the cognitive conflict that comes with the non-routine work of board, but also external pressures of contracts and regulative constraints, templates based in law or the practice of codes of conduct. Pressure is intensified by time constraints, deadlines and decisions to be made irrespective of the completeness of information available. But time plays a formative role as well. Directors come to know each other’s capabilities and the possibilities and limitations of the resource base.

The value arising from prolonged interaction is a reason that directors cite for resisting calls in codes for shorter director terms of office and for favoring staggered terms of office. The opposing argument, made from the shareholder side, is that longer, overlapping terms make both change and monitoring more difficult (Conference Board 2019; Govindarajan and Srivastava 2018). This suggests that, for directors, art-like elevation of sensibilities is unlikely to come from routine monitoring and control tasks but instead during tasks related to problem-solving and strategy formation associated with the service role of boards (Hillman and Dalziel 2003).

Most research into the strategy work of boards seeks either to link boards to outcomes (reviewed in Judge and Talaulicar 2017) or, in qualitative studies to understand the processes they use (Pettigrew and McNulty 1998; McNulty and Pettigrew 1999). Nonetheless, these studies and others provide glimpses of sources of director satisfaction that can arise. Consider, for example, the experience of consultant-like liminality in board work, in which directors actively sought informal ways to strategize when the pressure of routine compliance constrained the official board agenda in ways that blocked strategic discussion (Concannon and Nordberg 2018).

Working with the constraints, shaping the organization and its decisions to them, confers legitimacy (Cornforth and Edwards 1999), but following Dewey’s thinking, mere mimesis would represent a less elevating experience. Directors of the two professional organization boards studied by Parker (2007a) dealt with compliance and engaged in formal strategic planning. But they favored informal strategizing, often punctuated by “flashes of individual insights.” The more formal aspects of strategizing matched expectations of major stakeholders, enhancing legitimacy, while the informal aspect generated greater satisfaction.

Facing a strategic issue that imperiled one of the charities I serve, board meetings were still plagued by routine. We dispensed with that quickly, however, and made space in the agenda for free and frank discussions of the failures of prior approaches, the lessons to be drawn from them, and the generation of options for a way forward from the current predicament. That the discussions were frank led to pressure for action. That the actions did not yield the desired outcome by the deadlines led to further pressure but also deeper understanding of the internal weakness that needed to be addressed.

When the covid pandemic struck in 2020, both boards dispensed with much routine, empowering emergency committees to decide on responses to financial emergencies (at one, this proved not to be needed). Those taking part in these meetings, quickly became alert to details of the business and its operations in ways they had not followed, or needed to, in the more leisurely pace of ordinary business. We were unable to do very much at all; the power lay elsewhere, and luck played a big role. But we were connected. Those left off these committees, however, felt cut off, deprived of the experience.

In such board work, challenge to material presented by the executive is reflected against the outside experiences of non-executive directors and followed by the distillation of views into decisions. In the constantly shifting business environments of high-technology industries or social enterprises dependent upon powerful state buyers, decisions made once may need continual refinement and modification. Even when time was of the essence, decisions still take time and iteration. This points to a continuing need to return to the store of prior experiences and then back to the strategy canvas.

### Exciting the ‘Store’

For Dewey, the interaction of the internal and external, the compression involved in their integration, and the duration of the effort combine on a subject whose matter goes deep. When that happens, it can stir up “a store of attitudes and meanings derived from prior experience.” He likens this process to fire, which then inspires but also creates commotion and turmoil. It makes conscious thoughts, emotions, and images. That “fire” will either burn out or “press itself out … into a refined product…. Unless there is com-pression nothing is ex-pressed” (Dewey 1934/1958, punctuation in the original).

Much of the debate in the board meetings is routine, even perfunctory; it is the least likely to excite (ICSA 2011; Stiles and Taylor 2001; Parker 2007a). Decisions needs approval for reasons of constitutional formalities or because the value of a contract exceeds the agreed limit of managerial discretion. But those are not the decisions that directors remember, find satisfaction in having achieved, or regret for years to come. Board discussion of a proposed acquisition, a line of credit, or a regulatory action with implications for revenue, and how each would impact the risk calculus and reserves, involves debates that stir up meanings for other aspects of the business as well as memories of past decisions that have gone well or badly. These views are then synthesized, with parts rejected as less or irrelevant, in a word, compressed, pointing us toward the final expression. A multiple case study by Garg and Eisenhardt (2017) of boards of venture capital-backed businesses shows the importance of the dyadic relationship of the CEO and individual directors, but its selection of quotations also shows the excitement (or disappointment) about being included (or excluded) from discussions that matter.

In early 2020, as the covid-19 pandemic deepened and widened, the board of one of the organizations I serve held its final face-to-face meeting before the lockdown. We discussed the looming crisis, the steps that were needed and the ones that were closed to us, the financial and reputational risks, the way operations might adjust, the effects on beneficiaries, and potential ways to reconfigure service delivery. The board decided nothing; indeed, we felt helpless ahead of the storm. But the discussion led many of the directors to dig into their store of their experience for analogies, hints of what options might arise and how we might enact them. We each seemed to know that most and perhaps all of this would be useless, except perhaps in helping senior management anticipate the unexpected. When we had finished, one director smiled broadly as he left the room. “Good meeting!” he shouted in farewell to us all.

## The Experience of Observers

Dewey’s account of the nature of observer/audience experience of art is less fully articulated, but he draws a clear distinction between being receptive and passive. Receptivity is a process involving a “series of responsive acts that accumulate toward objective fulfillment” without which “there is not perception but [merely] recognition.” Experiencing a work of art is thus an active engagement with it, seeing the uniqueness of it, not the stereotypical. “The perceived object or scene is emotionally pervaded throughout,” he writes (1934/1958). That is, the emotions of the observer give meaning as much as those of the artist. “For to perceive, a beholder must *create* his own experience” (1934/1958, emphasis in the original). In this experience, the observer is the creator.

Corporate directors perform for a variety of audiences: investment analysts, shareholders, employees, even less well-defined ones during appearances on television. But these settings have little in common with the boardroom, where meetings take place with a high degree of confidentiality. Within the board, individual directors perform for each other, of course, though that “audience” is also an “actor.” Their experience is as much as that of a creator as it is of an observer. In the practice of corporate boards, the role of the audience in art is analogous to the presence of observers in board meetings. Academic literature on boards provides scant evidence of such events. Far more often, the board is called a “black box” from which observers are excluded (Klarner et al. 2020; Rost and Osterloh 2010). Researchers only occasionally penetrate it, and then to study directors (e.g. Pugliese et al. 2015), and not themselves.

There are, however, a few exceptions. A UK professional body examined what happens in meetings in large healthcare organizations when members of the public attend. Agendas and board discussions both avoided controversy and strategic debate, focusing instead on the routine (ICSA 2011). Knowing that they were being observed, these boards may have changed their behavior, however, and ethnographic accounts of boards are rare. Here I will draw on the anecdotal.

Visitors to the boards on which I have served talk about being there as a privilege, an intense experience of the complexity of decision-making and the scope of issues with which directors wrestled. These individuals were mainly middle managers unaccustomed to regular interactions with non-executive directors or executives acting in their role as directors. A few were “trainee directors,” individuals thought to have the potential to serve on the board and granted full access to board meetings and papers for a time, but without voting rights or legal responsibilities. Those enterprises are relatively simple businesses, with far fewer moving parts than a major listed company. Nonetheless visitors felt heightened awareness of business issues and gained insights they could not have experienced from outside. Visitors express what Dewey’s formulation would call perception, not just recognition.

## Discussion

Dewey’s ideas in aesthetics have not gone unnoticed in corporate governance. Pye (2001) notes that it helped her frame an eclectic study of boards. Still, there are at least two reasons to be cautious in stretching Dewey conceptualization into a seemingly unrelated field, especially with such limited evidence. First, Dewey’s is a formulation of its time and, in keeping with the pragmatist philosophy he espoused, it must be historically situated. Dewey might exclude much “high art” for its lack of emotion, but he relished jazz. Would he in old age have been happy with Los Angeles-style 1950s free jazz, or now with hip-hop? Second, even an intellectual friend like Shusterman finds Dewey’s aesthetics incomplete. Dewey’s distinction between expression and exposure, which relegates spontaneous explosions of emotion to a lesser state leads him (as observer) to disqualify Van Gogh’s creations as art. What would he have made Jackson Pollock? That is, even applied to its own field Dewey’s theory begs questions.

However, this paper does not assert theoretical completeness or claim discovery of a perfect analytic fit to the landscape of corporate governance. Nor does it assert that aesthetic experiences are frequent in boardrooms; indeed, the scant evidence in scholarly writing or practitioner accounts suggests it is not. Nor do such accounts allow us to think that an aesthetic view of governance could improve outcomes in terms of better firm performance or reduced risk. Instead, it seeks more modestly to suggest that our view of corporate governance may be enhanced by considering what excites directors in their work, and in so doing excite interest in exploring the experience of directors and corporate boards with fresh lenses.

Research that justifies governance activities by their outcomes takes an organizational and sometimes societal vantage point as to what matters. In so doing, it turns boards and directors into instruments of corporate performance and gives impetus to attempts to standardize structures and processes. When policy follows such guidance, it risks ignoring the humanist side of corporate governance, the motivation that leads people to engage in the effort. One aspect of that – and only one – may be the aesthetic aspects of board work.

### Implications for Practice of Corporate Governance

This paper asked the question, why would someone want to be a corporate director? Yes, the role comes with some glamor and external esteem, and often with some money. Even unpaid directorships can be stepping-stones to something more lucrative. But if the argument in this paper is valid, then those directors may wish to reflect on what holds their fellow directors’ attention. Many of the failings in corporate governance recorded in practitioner writing and in press accounts point the finger at directors or regulators who were absent or “asleep at the wheel” (Gribben 2008; see also MacAvoy and Millstein 2003). While some cases of a lack of watchfulness may be deliberate – directors choosing to ignore malfeasance or misdemeanors – the study of UK National Health Service boards illustrates how directors overlook important issues by the sheer tedium of the agenda (ICSA 2011). Keeping them alert requires an approach that commands their attention, which may be aided by seeing board work as a vehicle for expression. To be sure, much of board practice will remain routine, focused on the operational and compliance and lacking in emotional content. That is what in art Dewey calls “anesthetic.” But as the report on NHS governance by ICSA (2011) might have concluded, anesthetic governance can put you to sleep.

In the accounts above – from those few studies that penetrate the “black box” and from my reflections on events – we hear directors valorizing the intrinsic motivation that can come from the sense of an integrated whole that the work affords. There is also evidence of how the board provides a collective experience, building a social identity among the participants. Although it would be difficult to conduct such research, it would be helpful, for board practice and policy, to discover the conditions under which directors shake off the routine in favor of the aesthetic experience, but also the ways in which regular engagement with the routine prepares them for the extraordinary. As Dewey says, the artist experiences creation over time; self-expression is more than self-exposure. Heightened sensitivity may also arise among seemingly non-participating directors, those who listen quietly and say little. They may be observing now, while also building up a store that can be excited later, for example, in a boardroom coup that topples an incumbent but underperforming CEO.

### Implications for Corporate Governance Policy

Policy, too, can present a danger to the aesthetics of corporate governance. Focusing on outcomes-based analysis risks creating practices that reduce the work of directors to box-ticking (Fenwick and Vermeulen 2018; Rushton 2008), to mere compliance without reference to meaning-making (Filatotchev and Nakajima 2014). This is what Dewey might call a practice that fails to excite the “store” of meanings from experience. The work might lack the sense of integration that Parker (2007a) reports and that Dewey would see as an “experience” worthy of the name.

This argument does not diminish the importance of analysis in corporate governance, or of the need for psychic distance and challenge in the boardroom often associated with board effectiveness (Forbes and Milliken 1999; Nordberg and Booth 2019). Those aspects are often precisely what turns the practice of boards into an aesthetic experience for directors by showing the picture of the company in the round. Codes of conduct and regulation of practices like audit and corporate reporting can provide frameworks that directors see as elegant approaches to messy problems, as ways of seeing the whole among the pieces. But making compliance a mechanical exercise runs the risk of preventing directors perceiving the connections. Doing so risks depriving them of the sense of the integrated whole involved in the sort of decision-making from which they can derive satisfaction while also seeing issues in the round. For Dewey (1934/1958), “The mechanical stands at the pole opposite to that of the esthetic.”

### Limitations and Extensions

This analysis, viewing the experience of directors philosophically, as aesthetic satisfaction alongside its consequentialist instrumentality, is both empirically and conceptually narrow. The conceptual narrowness, a lens from a different domain in a rather different time, invites us to consider what other lenses might show. Even with this lens the narrowness might be overcome by examining another question: Does the “ugly” work of directors, the battles, the unresolved issues, the bust-ups over policy decisions that lead individual directors to the exit, also have an aesthetic quality? In the practice of the boards I have served, such events have also brought about heightened sensibilities, visions of the whole that seem to be slipping from our grasp. Not all dramas are comedies, after all; and the corporate world has experienced a fair few tragedies. Empirically, further research into director experiences of boardroom practice might include attempts to look beyond outcomes and processes into the psychological and symbolic elements of board work. This paper has scratched those surfaces by asking about director motivation and the meaning that physical boardrooms might bring to the experience of what takes within them. Under what conditions does “anesthetic” corporate governance arise?

There are reasons to believe these observations may not translate fully into the realm of large, listed companies, where other aspects of motivation apply. Non-executive directors in such companies also have material interests, at least in the form of director fees, and the ability to leverage board experience in one company to posts with others. Moreover, corporations can also arrange insurance to mitigate directors’ personal financial liability if not their reputational risk.^1^ Such firms have the resources to supply their boards with deeply researched proposals and finely crafted solutions presented to the board for ratification, potentially making board work more like dissection than creation. That is, the artistic experience may lie elsewhere in the organization, rather than with the board. Might these factors be reasons for big company boardroom ugliness?

## Conclusions

The artwork we observed at the opening of this paper is emblematic of something for each organization and the people who direct them. For one group, it was a sign of the success of the business, a decision of directors who saw themselves at the forefront of its industry, steeped in tradition (oak-paneled walls) but at the same to innovative and disruptive (impressionist, expressionist). For the other it was statement – by the board and senior management together – that the organization works for its beneficiaries. The objects themselves evidence directly benefits and the process of delivering them. These are reminders to anyone who enters the boardrooms of sort of experience they too should seek to achieve.

For Dewey, the “real work of art” is not the object created, but rather the psychic work it does for the artist and audience, “the building up of an integral experience out of the interaction of organic and environmental conditions and energies.” When the “excitement is deep” it stirs up meanings from prior experiences, which inform activities that become “conscious thoughts and emotions … To be set on fire by a thought or scene is to be inspired” (Dewey 1934/1958). In this Deweyan view, we may wish to consider the value of corporate governance to be more than just the outputs, not the statements in the annual report, and not even the profitability of a business decision. Value may lie as well in the experience of undertaking challenge and subsequent enlightenment, of constructing and reconstructing, over time, the basis of business decisions.

Using the language of his day, when the masculine included the feminine, Dewey tells a story of management:

Two men meet: one is the applicant for a position, while the other has the disposition of the matter is his hands. The interview may be mechanical, consisting of set questions, the replies to which perfunctorily settle the matter. … The situation is disposed of as if it were an exercise in bookkeeping. But an interplay may take place in which a new experience develops. Where should we look for an account of such an experience? Not in ledger-entries nor yet to a treatise on economics or sociology or personnel psychology, but to drama and fiction. Its nature can only be expressed in art, because there is a unity of experience that can be expressed only as an experience (Dewey 1934/1958).

In the study of corporate governance, we pay attention to ledger entries, economics, sociology, and psychology. We see – in academic studies and almost daily financial news media – evidence of the ugliness in its execution. In its practice, however, we sometimes experience its art. Perhaps we should study that, too.

In this small and tentative set of opinions and comments, the satisfaction participants in board meetings feel seems to arise through senses sharpened by boardroom debate, the experience of connecting many of the pieces of corporate decisions into a vision of the corporate whole. A board agenda that looks at the pieces but does not provide the opportunity to see the whole seems to present a different experience of being a director. If this argument is valid, policy too might benefit from seeking connections so those engaged in board practice might have a better chance of finding the art in corporate governance.

## Footnotes

1 The recent experience of the covid-19 pandemic illustrates the sense of personal risk that directors can face, notwithstanding mitigation through insurance. Anecdotal evidence suggests that several providers of directors’ and officers’ insurance withdrew from writing new policies in frontline sectors like social care or agreeing to increase coverage on existing policies. Brokers warned of large increases in premiums.
